# Evaluation of Turkey’s contribution to SCI-E indexed publications on COVID-19

**DOI:** 10.1186/s42077-022-00221-6

**Published:** 2022-03-10

**Authors:** Fulya Yılmaz, Koray Bas

**Affiliations:** 1 Department of Anaesthesiology and Reanimation, University of Health Sciences Izmir Bozyaka Training and Research Hospital, İzmir, Turkey; 2 Department of General Surgery, University of Health Sciences Izmir Bozyaka Training and Research Hospital, İzmir, Turkey

**Keywords:** COVID-19, 2019-n-CoV, SARS-CoV-2, Coronavirus disease 19, 2019 novel coronavirus, Turkey

## Abstract

**Background:**

In December of 2019, a new disease which is caused by SARS-CoV-2, as an epidemic disease out of Wuhan, China, began to circulate. On March 11, 2020, the Republic of Turkey Ministry of Health had announced the first case from Turkey. The aim of this study is to analyze the scientific publications in the field of COVID-19 included in Science Citation Index Expanded (SCIE) from Turkey and to establish a theoretical background for future studies in the health literature with obtained valuable information about the publications. We searched all papers published in the field of COVID-19 by using the terms of “COVID-19,” “2019-n-CoV,” “SARS-CoV-2,” “Coronavirus disease 19,” and “2019 novel coronavirus” as scientific nomenclatures of COVID-19 in the topic search section of the software.

**Results:**

Overall, 47,368 papers, indexed by SCI-E, were found related to COVID-19 between January 1, 2020, and December 13, 2020. Of these, 931 were from Turkey. In terms of specialities, the most contribution was from the Medicine General Internal followed by Dermatology. Most of the publications were article. English was the most preferred language in papers. *Dermatological Theraphy* published the most paper.

**Conclusions:**

Applying this kind of analysis on an intermittent basis gives a general perspective for contribution of a countries to scientific publications and useful for the further studies.

## Background

In December of 2019, a new disease which is caused by SARS-CoV-2, as an epidemic disease out of Wuhan, China, began to circulate (Gallegos et al., 2020). On March 11, 2020, World Health Organization (WHO) named this disease as “COVID-19” and declared “COVID-19” as a pandemic (Al-Zaman, n.d.). On the same date, the Republic of Turkey Ministry of Health had announced the first case from Turkey (Covid-19.saglik.gov.tr, n.d.)

Bibliometric researches indicate countries’ contribution toward scientific development (Yılmaz & Bas, 2020a). PubMed, Scopus, Web of Knowledge-Science (WoS, Thomson Reuters Company), and Google Scholar are well-known databases for bibliographic analysis (Yılmaz & Bas, 2020a; Yılmaz & Bas, 2020b). We preferred WoS, which is a popular database for data extraction for bibliometric analysis (https://covid19.saglik.gov.tr), was used for the search and the analysis.

Bibliometric analysis is defined as quantitative and/or qualitative analysis of certain characteristics of scientific documents or publications according to the researcher’s interest (Baş & Yılmaz, 2021a). It is an important indicator of scientific research productivity of countries contribution toward scientific development (Yılmaz & Bas, 2020a; Yılmaz & Bas, 2020b). To our knowledge, this study is the first bibliometric research from Turkey about COVID-19 on health.

The aim of this study is to analyze the scientific publications in the field of COVID-19 included in Science Citation Index Expanded (SCIE) from Turkey and to establish a theoretical background for future studies in the health literature with obtained valuable information about the publications.

## Methods

This search was performed in December 13, 2020, by using the WoS software to analyze COVID-19 publications included in the SCI-E. We retrospectively searched all publications published in the field of COVID-19 between January 1st and December 13th 2020 by using the terms of “COVID-19”, “2019-n-CoV”, “SARS-CoV-2”, “coronavirus disease 19” and “2019 novel coronavirus” as scientific nomenclatures of COVID-19 in the topic search section of the software. We applied an “Advanced Search” by using search operators as AND, OR, and NOT to reduce the risk of overlapping of papers in this time span.

Then by “analyze” function of the software, we analyzed the number of publications, web of science categories, type’s of papers, authors, name of journals, institutions, and citations for Turkey.

## Results

Overall, 47,368 papers, indexed by SCI-E, were found related to COVID-19 between January 1, 2020, and December 13, 2020. Of these 931 were from Turkey. The first paper published from Turkey was in April as a “short communication” included in SCIE publications.

In terms of specialities, according to science categories of WoS database, the most contribution was from Medicine General Internal (*n*=89), followed by dermatology (*n*=56), pharmacology pharmacy (*n*=53), psychiatry (*n*=50), and surgey (*n*=50). Others contribution were <50.

Most of the publications from Turkey were article (59.07%), followed by early access (24.38%), letter (17.61%), and review (14.39%) (Table 1). English (*n*=926) (99.46%) was the most preferred language in papers.

**Table 1:** Distribution in the types of papers published included in Science Citation Index Expanded, between January 1, 2020, and December 13, 2020, in the field of COVID-19

Document type	Number	Percentage
Article	550	59.07%
Early access	227	24.38%
Letter	164	17.61%
Review	134	14.39%
Editorial material	76	8.16%
Meeting abstract	6	0.64%
Correction	1	0.10%

Regarding authors’ contributions, Cure E and Cure MC were shared the first rank in the list (Table 2).

**Table 2:** Distribution of top 5 authors ranked by the number of papers published included in Science Citation Index Expanded, between January 1, 2020, and December 13, 2020, in the field of COVID-19

Rank	Author(s)	Number	Percentage
1	Cure E, Cure MC	16	59.07%
2	Lotti, T, Tursen U	12	24.38%
3	Karabay O	10	17.61%
4	Agache I, Bousquet J, Korkmaz S, Yaylacı S	9	14.39%
5	Akdis CA, Akdis M, Guclu E, Jutel M, Klimek L	8	8.16%

Journals that published ≥10 publications included in SCIE, between January 1, 2020, to December 13, 2020, in the field of COVID-19 are in Table 3. *Dermatological Theraphy* published the most paper. The distribution of the top 5 institutions which made up the highest percentages to contribute to the publications on COVID-19 from Turkey can be seen on Table 4. For those 931 publications, the sum of total citations during this time period was 2796, and the citation-to-work ratio was 3.

**Table 3:** Distribution of journals published ≥10 papers included in Science Citation Index Expanded, between January 1, 2020, and December 13, 2020, in the field of COVID-19

Rank	Journal	Number
1	Dermatological Theraphy	37
2	Journal of Medical Virology	27
3	Turkish Journal of Medical Sciences	20
4	Perspectives in Psychiatric Care	15
5	Revista Da Associacao Medica Brasileira	13
6	Allergy, Medical Hypotheses, Turkish Journal of Biology	12
7	International Journal of Clinical Practice	10

**Table 4:** Distribution of top 5 institutions ranked by the number of papers published included in Science Citation Index Expanded, between January 1, 2020, and December 13, 2020, in the field of COVID-19

Institution	Number
University of Health Sciences Turkey	119
Hacettepe University	63
Istanbul University	56
Ankara University	49
City Hospital Ankara	44

## Discussion

The most salient finding of our study was that the Dermatology rank the second raw in terms of specialities and the “Dermatological Theraphy” journal published the most paper in this time span.

In Turkey, the first publication on COVID-19 in SCI-E was published by Mandal H, as a “Short communication” in April 2020, on *Turkish Journal of Medical Sciences*. This publication was on “Mobilizing the research ecosystem for scientific advances toward positive impact in the context of the COVID-19 Pandemic”. Öztürk et al. (Ozturk, 2020) reported the trends of marketing literature during the COVID-19 pandemic in a review with bibliometric analysis from Turkey.

According to our analysis, Cure E and Cure MC shared the first rank as authors according to the number of publications in Turkey with the same publication. This paper was a letter that was published on July 2020, and on “Colchicine may not be effective in COVID-19; it may even be harmful?”. University of Health Sciences was ranked the first row among the institutions according to published papers. According to science categories of WoS database, the most contribution was from Medicine General Internal (*n*=89), followed by Dermatology (*n*=56) from Turkey. For journals, *Dermatological Theraphy* was the most preferred journal by authors from Turkey. In a study (Baş & Yılmaz, 2021b), the authors was analyzed the scientific activities in the first year of the COVID-19 pandemic overal the world. Although the “Dermatological Theraphy” journal rank the first in terms of publishing the papers according to our analyze; it was not take part in, in the top 10 journals that published the papers over the world.

According to science categories of WoS database in our analyze, Dematology rank the second raw and the dermatologist preferred “Dermatological Theraphy” journal to publish their papers. We may come up with a question like this: “Why dermatology journal published the most articles in Turkey?” We can explain this question according to the literature as follows:

1. In a study (Oba et al., 2021), the authors (dermatologists) searched the duties of dermatologists during COVID-19 pandemic in Turkey by an online 11-item questionnaire survey. They reported that while dermatologists in some countries (US,UK, Italy, Spain, and UK) were involved in the fight against COVID-19 pandemic in non-dermatology services including emergency department, inpatient wards, and intensive care units; in India and in Turkey, the dermatologists were not involved in the primary care of COVID-19 cases. They worked in dermatology wards that were converted to COVID wards. Only that 41.5–56.2% of dermatologists in Turkey were redeployed to pandemic inpatient clinics over 4 months at the beginning of the COVID-19.
2. In another study (Türsen et al., 2020), the dermatologists from Turkey stated that they were interested in COVID-19 management relevant to dermatologists, they discuss COVID-19 from dermatological aspect and they provide information for dermatology practice in this pandemic situation.
3. In another report (Karadag et al., 2021), the dermatologists mention “How their roles has changed in the COVID-19 pandemic”. They summarized them as to inform public about protective measures for cutaneous side effects associated with the intensive use of disinfectants and/or long-term use of masks, the issues with teledermatology and online education.
4. In a viewpoint (Shinkai et al., 2020), dermatologists thought that their important role in COVID-19 pandemic was to continue to learn and contribute, determine whether cutaneous manifestations were signal important systemic associations, and consider how they can best diagnose and safely and effectively treat patients with COVID-19–related skin manifestations while retaining the highest quality for all patients who require dermatologic care. So they contributed to the literature with case reports, small case series, and opinion articles.

The limitation of this study is the WoS program we used in our research cannot report the publications month by month. So we cannot analyze the changes of publication types and number of publications of the countries month by month. Therefore, we cannot report the changes in publication types of countries, and we cannot comment on the accelerations of countries in publications month by month.

## Conclusions

Applying this kind of analysis on an intermittent basis gives a general perspective for contribution of a countries to scientific publications and useful for the further studies.

## Data Availability

The datasets used and/or analyzed during the current study are available from the corresponding author on reasonable request.

## Abbreviations

SCI-E: Science Citation Index Expanded
WHO: World Health Organization
WoS: Web of Knowledge-Science
